# Hammer for Coq: Automation for Dependent Type Theory

**DOI:** 10.1007/s10817-018-9458-4

**Published:** 2018-02-27

**Authors:** Łukasz Czajka, Cezary Kaliszyk

**Affiliations:** University of Innsbruck, Innsbruck, Austria

**Keywords:** Hammer, Coq, Calculus of inductive constructions, Proof automation

## Abstract

Hammers provide most powerful general purpose automation for proof assistants based on HOL and set theory today. Despite the gaining popularity of the more advanced versions of type theory, such as those based on the Calculus of Inductive Constructions, the construction of hammers for such foundations has been hindered so far by the lack of translation and reconstruction components. In this paper, we present an architecture of a full hammer for dependent type theory together with its implementation for the Coq proof assistant. A key component of the hammer is a proposed translation from the Calculus of Inductive Constructions, with certain extensions introduced by Coq, to untyped first-order logic. The translation is “sufficiently” sound and complete to be of practical use for automated theorem provers. We also introduce a proof reconstruction mechanism based on an eauto-type algorithm combined with limited rewriting, congruence closure and some forward reasoning. The algorithm is able to re-prove in the Coq logic most of the theorems established by the ATPs. Together with machine-learning based selection of relevant premises this constitutes a full hammer system. The performance of the whole procedure is evaluated in a bootstrapping scenario emulating the development of the Coq standard library. For each theorem in the library only the previous theorems and proofs can be used. We show that 40.8% of the theorems can be proved in a push-button mode in about 40 s of real time on a 8-CPU system.

## Introduction

*Interactive Theorem Proving* (ITP) systems [44] become more important in certifying mathematical proofs and properties of software and hardware. A large part of the process of proof formalisation consists of providing justifications for smaller goals. Many of such goals would be considered trivial by mathematicians. Still, modern ITPs require users to spend an important part of the formalisation effort on such easy goals. The main points that constitute this effort are usually library search, minor transformations on the already proved theorems (such as reordering assumptions or reasoning modulo associativity-commutativity), as well as combining a small number of simple known lemmas.

ITP automation techniques are able to reduce this effort significantly. Automation techniques are most developed for systems that are based on somewhat simple logics, such as those based on first-order logic, higher-order logic, or the untyped foundations of ACL2. The strongest general purpose proof assistant automation technique is today provided by tools called “hammers” [17] which combine learning from previous proofs with translation of the problems to the logics of automated systems and reconstruction of the successfully found proofs. For many higher-order logic developments a third of the proofs can be proved by a hammer in push-button mode [15, 52].

Even if the more advanced versions of type theory, as implemented by systems such as Agda [13], Coq [14], Lean [29], and Matita [5], are gaining popularity, there have been no hammers for such systems. This is because building such a tool requires a usable encoding, and a strong enough proof reconstruction.

A typical use of a hammer is to prove relatively simple goals using available lemmas. The problem is to find appropriate lemmas in a large collection of all accessible lemmas and combine them to prove the goal. An example of a goal solvable by our hammer, but not solvable by any standard Coq tactics, is the following. 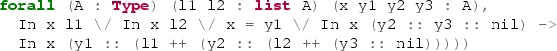 The statement asserts that if x occurs in one of the lists l1, l2, or it is equal to y1, or it occurs in the list 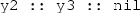 consisting of the elements y2 and y3, then it occurs in the list 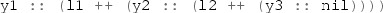 where ++ denotes list concatenation and :: denotes the list cons operator. Eprover almost instantly finds a proof of this goal using six lemmas from the module Lists.List in the Coq standard library:
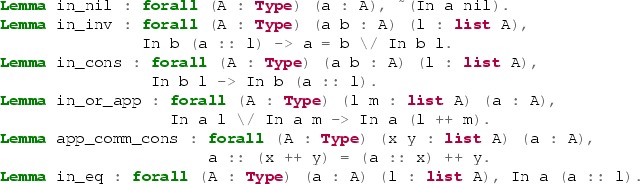
The found ATP proof may be automatically reconstructed inside Coq.

The advantage of a hammer is that it is a general system not depending on any domain-specific knowledge. The hammer plugin may use all currently accessible lemmas, including those proven earlier in a given formalization, not only the lemmas from the standard library or other predefined libraries.

*Contributions*. In this paper we present a comprehensive hammer for the Calculus of Inductive Constructions together with an implementation for the Coq proof assistant. In particular:

- We introduce an encoding of the Calculus of Inductive Constructions, including the additional logical constructions introduced by the Coq system, in untyped first-order logic with equality.
- We implement the translation and evaluate it experimentally on the standard library of the Coq proof assistant showing that the encoding is sufficient for a hammer system for Coq: the success rates are comparable to those demonstrated by hammer systems for Isabelle/HOL and Mizar, while the dependencies used in the ATP proofs are most often sufficient to prove the original theorems.
- We present a proof reconstruction mechanism based on an eauto-type procedure combined with some forward reasoning, congruence closure and heuristic rewriting. Using this proof search procedure we are able to re-prove 44.5% of the problems in the Coq standard library, using the dependencies extracted from the ATP output.
- The three components are integrated in a plugin that offers a Coq automation tactic hammer. We show case studies how the tactic can help simplify certain existing Coq proofs and prove some lemmas not provable by standard tactics available in Coq.

Preliminary versions of the translation and reconstruction components for a hammer for Coq have been presented by us at HaTT 2016 [24]. Here, we improve both, as well as introduce the other required components creating a first whole hammer for a system based on the Calculus of Inductive Constructions.

The rest of this paper is structured as follows. In Sect. 2 we discuss existing hammers for other foundations, as well as existing automation techniques for variants of type theory including the Calculus of Constructions. In Sect. 3 we introduce ${\mathrm {CIC}}_0$, an approximation of the Calculus of Inductive Constructions which will serve as the intermediate representation for our translation. Section 4 discusses the adaptation of premise selection to ${\mathrm {CIC}}_0$. The two main contribution follow: the translation to untyped first-order logic (Sect. 5) and a mechanism for reconstructing in Coq the proofs found by the untyped first-order ATPs 6. The construction of the whole hammer and its evaluation is given in Sect. 7. Finally in Sect. 8 a number of case studies of the whole hammer is presented.

## Related Work

A recent overview [17] discusses the three most developed hammer systems, large-theory premise selection, and the history of bridges between ITP and ATP systems. Here we briefly survey the architectures of the three existing hammers and their success rates on the various considered corpora, as well as discuss other related automation techniques for systems based on the Calculus of (Inductive) Constructions.

### Existing Hammers

Hammers are proof assistant tools that employ external automated theorem provers (ATPs) in order to automatically find proofs of user given conjectures. Most developed hammers exist for proof assistants based on higher-order logic (Sledgehammer [63] for Isabelle/HOL [74], HOLyHammer [52] for HOL Light [40] and HOL4 [67]) or dependently typed set theory (MizAR [55] for Mizar [10, 73]). Less complete tools have been evaluated for ACL2 [46]. There are three main components of such hammer systems: premise selection, proof translation, and reconstruction.

Premise Selection is a module that given a user goal and a large fact library, predicts a smaller set of facts likely useful to prove that goal. It uses the statements and the proofs of the facts for this purpose. Heuristics that use recursive similarity include SInE [45] and the Meng-Paulson relevance filter [62], while the machine-learning based algorithms include sparse naive Bayes [70] and *k*-nearest neighbours (k-NN) [51]. More powerful machine learning algorithms perform significantly better on small benchmarks [1], but are today too slow to be of practical use in ITPs [34, 58].

Translation (encoding) of the user given conjecture together with the selected lemmas to the logics and input formats of automated theorem provers (ATPs) is the focus of the second module. The target is usually first-order logic (FOL) in the TPTP format [68], as the majority of the most efficient ATPs today support this foundation and format. Translations have been developed separately for the different logics of the ITPs. An overview of the HOL translation used in Sledgehammer is given in [18]. An overview of the dependently-typed set theory of MizAR is given in  [72]. The automated systems are in turn used to either find an ATP proof or just further narrow down the subset of lemmas to precisely those that are necessary in the proof (unsatisfiable core).

Finally, information obtained by the successful ATP runs can be used to re-prove the facts in the richer logic of the proof assistants. This is typically done in one of the following three ways. First, by a translation of the found ATP proof to the corresponding ITP proof script [9, 64], where in some cases the script may be even simplified to a single automated tactic parametrised by the used premises. Second, by replaying the inference inside the proof assistant [20, 50, 64]. Third, by implementing verified ATPs [3], usually with the help of code reflection.

The general-purpose automation provided by the most advanced hammers is able to solve 40–50% of the top-level goals in various developments [17], as well as more than 70% of the user-visible subgoals [15].

### Related Automation Techniques

The encodings of the logics of proof assistants based on the Calculus of Constructions and its extensions in first-order logic have so far covered only very limited fragments of the source logic [2, 16, 69]. Why3 [35] provides a translation from its own logic [33] (which is a subset of the Coq logic, including features like rank-1 polymorphism, algebraic data types, recursive functions and inductive predicates) to the format of various first-order provers (in fact Why3 has been initially used as a translation back-end for HOLyHammer).

Certain other components of a hammer have already been explored for Coq. For premise selection, we have evaluated the quality of machine learning advice [49] using custom implementations of Naive Bayes relevance filter, k-Nearest Neighbours, and syntactic similarity based on the Meng-Paulson algorithm [62]. *Coq Learning Tools* [59] provides a user interface extension that suggests to the user lemmas that are most likely useful in the current proof using the above algorithms as well as LDA. The suggestions of tactics which are likely to work for a given goal has been attempted in ML4PG [48], where the Coq Proof General [6] user interface has been linked with the machine learning framework Weka [41]. SEPIA [39] tries to infer automata based on existing proofs that are able to propose likely tactic sequences.

The already available HOL automation has been able to reconstruct the majority of the automatically found proofs using either internal proof search [43] or source-level reconstruction. The internal proof search mechanisms provided in Coq, such as the firstorder tactic [26], have been insufficient for this purpose so far: we will show this and discuss the proof search procedures of firstorder and tauto in Sect. 6. The jp tactic which integrates the intuitionistic first-order automated theorem prover JProver [66] into Coq does not achieve sufficient reconstruction rates either [24]. Matita’s ordered paramodulation [7] is able to reconstruct many goals with up to two or three premises, and the congruence-closure based internal automation techniques in Lean [30] are also promising.

The SMTCoq [3] project has developed an approach to use external SAT and SMT solvers and verify their proof witnesses. Small checkers are implemented using reflection for parts of the SAT and SMT proof reconstruction, such as one for CNF computation and one for congruence closure. The procedure is able to handle Coq goals in the subset of the logic that corresponds to the logics of the input systems.

## Type Theory Preliminaries

In this section we present our approximation ${\mathrm {CIC}}_0$ of the Calculus of Inductive Constructions, i.e., of the logic of Coq. The system ${\mathrm {CIC}}_0$ will be used as an intermediate step in the translation, as well as the level at which premise selection is performed. Note that ${\mathrm {CIC}}_0$ is interesting as an intermediate step in the translation, but is not a sound type theory by itself (this will be discussed in Sect. 5.6). We assume the reader to be familiar with the Calculus of Constructions [22] and to have a working understanding of the type system of Coq [11, 25]. This section is intended to fix notation and to precisely define the syntax of the formalism we translate to first-order logic. The system ${\mathrm {CIC}}_0$ is intended as a precise description of the syntax of our intermediate representation. It is a substantial fragment of the logic of Coq as presented in [25, Chapter 4], as well as of other systems based on the Calculus of Constructions. The features of Coq not represented in the formalism of ${\mathrm {CIC}}_0$ are: modules and functors, coinductive types, primitive record projections, and universe constraints on ${\mathrm {Type}}$.

The formalism of ${\mathrm {CIC}}_0$ could be used as an export target for other proof assistants based on the Calculus of Inductive Constructions, e.g. for Matita or Lean. However, in ${\mathrm {CIC}}_0$, like in Coq, Matita and Lean, there is an explicit distinction between the universe of propositions ${\mathrm {Prop}}$ and the universe of sets ${\mathrm {Set}}$ or types ${\mathrm {Type}}$. The efficiency of our translation depends on this distinction: propositions are translated directly to first-order formulas, while sets or types are represented by first-order terms. For proof assistants based on dependent type theories which do not make this distinction, e.g. Agda [13] and Idris [19], one would need a method to heuristically infer which types are to be regarded as propositions, in addition to possibly some adjustments to the formalism of ${\mathrm {CIC}}_0$.

The language of ${\mathrm {CIC}}_0$ consists of terms and three forms of declarations. First, we present the possible forms of terms of ${\mathrm {CIC}}_0$ together with a brief intuitive explanation of their meaning. The terms of ${\mathrm {CIC}}_0$ are essentially simplified terms of Coq. Below by *t*, *s*, *u*, $\tau$, $\sigma$, $\rho$, $\kappa$, $\alpha$, $\beta$, etc., we denote terms of ${\mathrm {CIC}}_0$, by *c*, $c'$, *f*, *F*, etc., we denote constants of ${\mathrm {CIC}}_0$, and by *x*, *y*, *z*, etc., we denote variables. We use $\vec {t}$ for a sequence of terms $t_1 \ldots t_n$ of an unspecified length *n*, and analogously for a sequence of variables $\vec {x}$. For instance, $s \vec {y}$ stands for $s y_1 \ldots y_n$, where *n* is not important or implicit in the context. Analogously, we use $\lambda \vec {x} : \vec {\tau } . t$ for $\lambda x_1 : \tau _1 . \lambda x_2 : \tau _2 . \ldots \lambda x_n : \tau _n . t$, with *n* implicit or unspecified.

A *term* of ${\mathrm {CIC}}_0$ has one of the following forms.

- *c*. A constant.
- *x*. A variable.
- *ts*. An application.
- $$\lambda x : t . s$$. A lambda-abstraction.
- $\varPi x : t . s$. A dependent product. If *x* does not occur free in *s* then we abbreviate $\varPi x : t . s$ by $t \rightarrow s$.
- ${\mathtt {case}}(t, c, n, \lambda \vec {a} : \vec {\alpha } . \lambda x : c \vec {p} \vec {a} . \tau , \lambda \vec {x_1} : \vec {\tau _1} . s_1, \ldots , \lambda \vec {x_k} : \vec {\tau _k} . s_k)$. A case expression. Here *t* is the term matched on, *c* is a constant such that
  $$\begin{aligned} I_n(c : \gamma {:}{=} c_1 : \gamma _1, \ldots , c_k : \gamma _k) \end{aligned}$$
  is an inductive declaration in the global environment (see the definition of inductive declarations below for an explanation), the type of *t* has the form $c \vec {p} \vec {u}$, the integer *n* denotes the number of parameters (which is the length of $\vec {p}$), the type $\tau [\vec {u}/\vec {a},t/x]$ is the return type, i.e., the type of the whole case expression, $\vec {a} \cap {\mathrm {FV}}(\vec {p}) = \emptyset$, and $s_i[\vec {v}/\vec {x_i}]$ is the value of the case expression if the value of *t* is $c_i \vec {p} \vec {v}$.
- ${\mathtt {fix}}(f_i, f_1 : t_1 {:}{=} s_1, \ldots , f_n : t_n {:}{=} s_n)$. A mutually recursive fixpoint definition. The value of this is the function $f_i$ (where $1 \le i \le n$) defined by $s_i$. The variables $f_1,\ldots ,f_n$ may occur in $s_1,\ldots ,s_n$. All functions are required to be terminating.
- ${\mathtt {let}}(x : t {:}{=} s, u)$. A let-expression locally binding *x* of type *t* to *s* in *u*.
- ${\mathtt {cast}}(t, \tau )$. A type cast: *t* is forced to have type $\tau$.

We assume that the following special constants are among the constants of ${\mathrm {CIC}}_0$: ${\mathrm {Prop}}$, ${\mathrm {Set}}$, ${\mathrm {Type}}$, $\top$, $\bot$, $\forall$, $\exists$, $\wedge$, $\vee$, $\leftrightarrow$, $\lnot$, $=$. We usually write $\forall x : t . s$ and $\exists x : t . s$ instead of $\forall t (\lambda x : t . s)$ and $\exists t (\lambda x : t . s)$, respectively. For $\wedge$, $\vee$ and $\leftrightarrow$ we typically use infix notation. We usually write $t = s$ instead of $= \tau s t$, omitting the type $\tau$. The purpose of having the *logical primitives* $\top , \bot , \forall , \exists , \wedge , \vee , \leftrightarrow , \lnot , {=}$ in ${\mathrm {CIC}}_0$ is to be able to directly represent the Coq definitions of logical connectives. These primitives are used during the translation. We directly export the Coq definitions and inductive types which represent the logical connectives (the ones declared in the Init.Logic module), as well as equality, to the logical primitives of ${\mathrm {CIC}}_0$. In particular, Init.Logic.all is exported to $\forall$.

In ${\mathrm {CIC}}_0$ the universe constraints on ${\mathrm {Type}}$ present in the Coq logic are lost. This is not dangerous in practice, because the ATPs are not strong enough to exploit the resulting inconsistency. Proofs of paradoxes present in Coq’s standard library are explicitly filtered-out by our plugin.

A *declaration* of ${\mathrm {CIC}}_0$ has one of the following forms.

- A *definition* $c = t : \tau$. This is a definition of a constant *c* stating that *c* is (definitionally) equal to *t* and it has type $\tau$.
- A *typing declaration* $c : \tau$. This is a declaration of a constant *c* stating that it has type $\tau$.
- An *inductive declaration* $I_k(c : \tau {:}{=} c_1 : \tau _1, \ldots , c_n : \tau _n)$ of *c* of type $\tau$ with *k* parameters and *n* constructors $c_1,\ldots ,c_n$ having types $\tau _1,\ldots ,\tau _n$ respectively. We require $\tau \Downarrow \varPi \vec {y} : \vec {\sigma } . \varPi \vec {y}' : \vec {\sigma }'. s$ with $s \in \{{\mathrm {Prop}},{\mathrm {Set}},{\mathrm {Type}}\}$ and $\tau _i \Downarrow \varPi \vec {y} : \vec {\sigma } . \vec {x_i} : \vec {\alpha _i} . c \vec {y} \vec {u_i}$ for $i=1,\ldots ,n$, where the length of $\vec {y}$ is *k* and $a \Downarrow b$ means that *a* evaluates to *b*. Usually, we omit the subscript *k* when irrelevant or clear from the context. For instance, a polymorphic type of lists defined as an inductive type in ${\mathrm {Type}}$ with a single parameter of type ${\mathrm {Type}}$ may be represented by
  $$\begin{aligned} \begin{array}{l} I_1(\mathtt {List} : {\mathrm {Type}}\rightarrow {\mathrm {Type}}{:}{=}\\ \quad \quad \quad \mathtt {nil} : (\varPi A : {\mathrm {Type}}. \mathtt {List}\,A),\\ \quad \quad \quad \mathtt {cons} : (\varPi A : {\mathrm {Type}}. A \rightarrow \mathtt {List}\, A \rightarrow \mathtt {List}\,A)). \end{array} \end{aligned}$$
  Mutually inductive types may also be represented, because we do not require the names of inductive declarations to occur in any specific order. For instance, the inductive predicates $\mathtt {even}$ and $\mathtt {odd}$ may be represented by two inductive declarations
  $$\begin{aligned} \begin{array}{l} I_0(\mathtt {even} : \mathtt {nat} \rightarrow {\mathrm {Prop}}{:}{=}\\ \quad \quad \quad \mathtt {even\_0} : \mathtt {even}\, 0,\\ \quad \quad \quad \mathtt {even\_S} : \varPi n : \mathtt {nat} . \mathtt {odd}\,n \rightarrow \mathtt {even}\, (S n)). \\ I_0(\mathtt {odd} : \mathtt {nat} \rightarrow {\mathrm {Prop}}{:}{=}\\ \quad \quad \quad \mathtt {odd\_S} : \varPi n : \mathtt {nat} . \mathtt {even}\,n \rightarrow \mathtt {odd}\, (S n)). \end{array} \end{aligned}$$

An *environment* of ${\mathrm {CIC}}_0$ is a set of declarations. We assume an implicit global environment *E*. The environment *E* is assumed to contain appropriate typing declarations for the logical primitives. A ${\mathrm {CIC}}_0$ *context* is a list of declarations of the form *x* : *t* with *t* a term of ${\mathrm {CIC}}_0$ and *x* the declared ${\mathrm {CIC}}_0$ variable. We assume the variables declared in a context are pairwise disjoint. We denote environments by *E*, $E'$, etc., and contexts by $\varGamma$, $\varGamma '$, etc. We write $\varGamma , x : \tau$ to denote the context $\varGamma$ with $x : \tau$ appended. We denote the empty context by $\langle \rangle$. A type judgement of ${\mathrm {CIC}}_0$ has the form $\varGamma \vdash t : \tau$ where $\varGamma$ is a context and $t,\tau$ are terms. If $\varGamma \vdash t : \tau$ and $\varGamma \vdash \tau : \sigma$ then we write $\varGamma \vdash t : \tau : \sigma$. A $\varGamma$*-proposition* is a term *t* such that $\varGamma \vdash t : {\mathrm {Prop}}$. A $\varGamma$*-proof* is a term *t* such that $\varGamma \vdash t : \tau : {\mathrm {Prop}}$ for some term $\tau$.

The set ${\mathrm {FV}}(t)$ of free variables of a term *t* is defined in the usual way. To save on notation we sometimes treat ${\mathrm {FV}}(t)$ as a list. For a context $\varGamma$ which includes declarations of all free variables of *t*, the free variable context ${\mathrm {FC}}(\varGamma ;t)$ of *t* is defined inductively:

- $${\mathrm {FC}}(\langle \rangle ; t) = \langle \rangle $$,
- $${\mathrm {FC}}(\varGamma , x : \tau ; t) = {\mathrm {FC}}(\varGamma ; \lambda x : \tau . t), x : \tau $$ if $$x \in {\mathrm {FV}}(t)$$,
- $${\mathrm {FC}}(\varGamma , x : \tau ; t) = {\mathrm {FC}}(\varGamma ; t)$$ if $$x \notin {\mathrm {FV}}(t)$$.

If $\varGamma$ includes declarations of all variables from a set of variables *V*, then we define ${\mathrm {FF}}_\varGamma (V)$ to be the set of those $y \in V$ which are not $\varGamma$-proofs. Again, to save on notation we sometimes treat ${\mathrm {FF}}_\varGamma (V)$ as a list.

Our translation encodes ${\mathrm {CIC}}_0$ in untyped first-order logic with equality (FOL). We also implemented a straightforward information-forgetting export of Coq declarations into the syntax of ${\mathrm {CIC}}_0$. We describe the translation and the export in the next section.

In the translation of ${\mathrm {CIC}}_0$ we need to perform (approximate) type checking to determine which terms are propositions (have type ${\mathrm {Prop}}$), i.e. we need to check whether a given term *t* in a given context $\varGamma$ has type ${\mathrm {Prop}}$. For this purpose we implemented a specialised efficient procedure to do so. In fact, this procedure is slightly incomplete. The point here is to approximately identify which types are intended to represent propositions. In proof assistants or proof developments where types other than those of sort ${\mathrm {Prop}}$ are intended to represent propositions the procedure needs to be changed.

All ${\mathrm {CIC}}_0$ terms we are interested in correspond to typable (and thus strongly normalizing) Coq terms, i.e., Coq terms are exported in a simple information-forgetting way to appropriate ${\mathrm {CIC}}_0$ terms. We will assume that for any exported term there exists a type in logic of Coq, it is unique, and it is preserved under context extension. This assumption is not completely theoretically justified, but is useful in practice.

## Premise Selection

The first component of a hammer preselects a subset of the accessible facts most likely to be useful in proving the user given goal. In this section we present the premise selection algorithm proposed for a hammer for dependently typed theory. We reuse the two most successful filters used in HOLyHammer [52] and Sledgehammer [15] adapted to the ${\mathrm {CIC}}_0$ representation of proof assistant knowledge. We first discuss the features and labels useful for that representation and further describe the *k*-NN and naive Bayes classifiers, which we used in our implementation.

### Features and Labels

A simple possible characterization of statements in a proof assistant library is to use the sets of symbols that appear in these statements. It is possible to extend this set in many ways [56], including various kinds of structure of the statements, types, and normalizing variables (all variables will be replaced by a single symbol X). In the case of ${\mathrm {CIC}}_0$, the constants are already both term constants and type constructors. We omit the basic logical constants, as they will not be useful for automated theorem provers which assume first-order logic. We further augment the set of features by inspecting the parse tree: constants and constant-variable pairs that share an edge in the parse tree give rise to a feature of the statement. We will denote such features of a theorem *T* by *F*(*T*).

For each feature *f* we additionally compute a feature weight *w*(*f*) that estimates the importance of the feature. Based on the HOLyHammer experiments with feature weights [54], we use TF-IDF [47] to compute feature weights. This ensures that rare features are more important than common ones.

Like in usual premise selection, the dependencies of theorems will constitute the labels for the learning algorithms. The dependencies for a theorem or definition *T*, which we will denote *D*(*T*), are the constants occuring in the type of *T* or in the proof term (or the unfolding) of *T*. Note that these dependencies may not be complete, because in principle an ATP proof of *T* may need some additional information that in Coq is incorporated into type-checking but not used to build proof terms, e.g. definitions of constants, facts which are necessary to establish types of certain terms.

For example, consider the theorem $T = {\texttt {Between.between\_le}}$ from the Coq standard library with the statement: 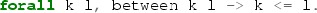 In the section where this theorem is declared there is the following variable declaration: 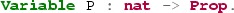 The features and dependencies of *T* are:

$$\begin{aligned} F(T)= & {} \{{\texttt {"Between.Between.between","Between.Between.between-X",}} \\&{\texttt {"Coq.Init.Datatypes.nat", "Coq.Init.Peano.le", }}\\&{\texttt {"Coq.Init.Peano.le-X"}}\} \\ D(T)= & {} \{{\texttt {"Between.Between.between","Between.Between.between\_ind",}} \\&{\texttt {"Coq.Init.Datatypes.nat", "Coq.Init.Peano.le",}} \\&{\texttt {"Coq.Init.Peano.le\_S", "Coq.Init.Peano.le\_n", "P"}}\} \\ \end{aligned}$$

The -X features correspond to constants applied to variables. Similarly, in more complex examples constant-constant applications (such as the successor of zero) give rise to such compound features.

### *k*-Nearest Neighbors

The *k* nearest neighbors classifier (*k*-NN) finds a given number *k* of accessible facts which are most similar to the current goal. The distance for two statements *a*, *b* is defined by the function (higher values means more similar, $\tau _1$ is a constant which gives more similar statements an additional advantage):

$$\begin{aligned} s(a, b) = {\sum \limits _{\,f \in F(a) \cap F(b)}{w(f)^{\tau _1}}} \end{aligned}$$

The dependencies of the selected facts will be used to estimate the relevance of all accessible facts. Given the set of the *k* nearest neighbors *N* together with their nearness values, the relevance of a visible fact $a$ for the goal *g* is

$$\begin{aligned} \left( \tau _2 \sum \limits _{b\in N \mid a\in D(b)\,} \frac{s(b, g)}{\left| D(b)\right| }\right) \mathrel + {{\left\{ \begin{array}{ll} s(a, g) &{} \text {if } a\in N \\ 0 &{} \text {otherwise} \end{array}\right. }} \end{aligned}$$

where $\tau _2$ is a constant which gives more importance to the dependencies. We have used the values $\tau _1 = 6$ and $\tau _2 = 2.7$ in our implementation, which were found experimentally in our previous work [51].

There are two modifications of the standard *k*-NN algorithm. First, when deciding on the labels to predict based on the neighbors, we not only include the labels associated with the neighbors based on the training examples (this corresponds to past proofs) but also the neighbors themselves. This is because a theorem is in principle provable from itself in zero steps, and this information is not included in the training data. Furthermore, theorems that have been proved, but have not been used yet, would not be accessible to the algorithm without this modification.

Second, we do not use a fixed number *k*, instead we fix the number of facts with non-zero relevance that need to be predicted. We start with $k=1$ and if not enough facts have been selected, we increase *k* iteratively. This allows creating ATP problems of proportionate complexity.

### Sparse Naive Bayes

The sparse naive Bayes classifier estimates the relevance of a fact $a$ for a goal *g* by the probability

$$\begin{aligned} P(a\text { is used in the proof of } g ) \end{aligned}$$

Since the goal is only characterized by its features, the probability can be further estimated by:

$$\begin{aligned} P(a\text { is used in a proof of } s \mid s \text { has features } F(g) ) \end{aligned}$$

where *s* is an arbitrary proved theorem, abstracting from the goal *g*.

For efficiency reasons the computation of the relevance of *a* is restricted to the features of *a* and the features that were ever present when *a* was used as a dependency. More formally, the *extended features* $\overline{F}(a)$ of $a$ are:

$$\begin{aligned} \overline{F}(a) = F(a) \cup \bigcup _{a\in {\mathrm {D}}(b)} \,F(b) \end{aligned}$$

The probability can be thus estimated by the statements *s* which have the features *F*(*g*) but do not have the features $\overline{F}(a) - F(g)$:

$$\begin{aligned} P\bigl (a\text { is used in a proof of } s \mid F(a) \subseteq F(g) \wedge F(a) \text { misses } \overline{F}(a) - F(g) \bigr ) \end{aligned}$$

Assuming that the features are independent^1^ the Bayes’s rule can be applied to transform the probability to the following product of probabilities:

$$\begin{aligned}&P(a\text { is used in the proof of } s)\\&\quad \mathrel \cdot \prod \limits _{f\in F(g)\cap \overline{F}(a)\,} P\bigl (s \text { has feature } f \mid a\text { is used in the proof of } s\bigr ) \\&\quad \mathrel \cdot \prod \limits _{f\in F(g)-\overline{F}(a)\,} P\bigl (s \text { has feature } f \mid a\text { is not used in the proof of } s\bigr ) \\&\quad \mathrel \cdot \prod \limits _{f\in \overline{F}(a)-F(g)\,} P\bigl (s \text { does not have feature } f \mid a\text { is used in the proof of } s\bigr ) \end{aligned}$$

The expressions can be finally estimated:

$$\begin{aligned}&P(a\text { is used in a proof of } s) = \frac{\smash {t(a)}}{K} \\&P\bigl (s \text { has feature } f \mid a\text { is used in the proof of } s\bigr ) = \frac{s(a, f)}{t(a)}\\&P\bigl (s \text { does not have feature } f \mid a\text { is used in the proof of } s\bigr ) = 1 - \frac{s(a, f)}{t(a)} \end{aligned}$$

using two auxiliary functions that can be computed from the dependencies:

- $s(a,f)$ is the number of times $a$ has been a dependency of a fact characterized by the feature *f*;
- $t(a)$ is the number of times $a$ has been a dependency;

as well as the number *K* of all theorems proved so far.

In our actual implementation we further introduce minor modifications to avoid any of the probabilities become zero and we estimate the logarithms of probabilities to avoid multiplying small numbers which might cause numerical instability. The classifier can finally estimate the relevance of all visible facts and return the requested number of them that are most likely to lead to a successful proof of the conjecture.

## Translation

In this section we describe a translation of Coq goals through ${\mathrm {CIC}}_0$ to untyped first-order logic with equality. The translation presented here is a significantly improved version of our translation presented at HaTT [24]. It has been made more complete, many optimisations have been introduced, and several mistakes have been eliminated.

The translation is neither sound nor complete. In particular, it assumes proof irrelevance (in the sense of erasing proof terms), it omits universe constraints on ${\mathrm {Type}}$, and some information is lost in the export to ${\mathrm {CIC}}_0$. However, it is sound and complete “enough” to be practically usable by a hammer (just like the hammers for other systems, it works very well for essentially first-order logic goals and becomes much less effective with other features of the logics [17]). The limitations of the translation and further issues of the current approach are explained in more detail in Sects. 5.6 and 9. Some similar issues were handled in the context of code extraction in [60].

The translation proceeds in three phases. First, we export Coq goals to ${\mathrm {CIC}}_0$. Next we translate ${\mathrm {CIC}}_0$ to first-order logic with equality. In the first-order language we assume a unary predicate *P*, a binary predicate *T* and a binary function symbol @. Usually, we write *ts* instead of @(*t*, *s*). Intuitively, an atom of the form *P*(*t*) asserts the provability of *t*, and $T(t,\tau )$ asserts that *t* has type $\tau$. In the third phase we perform some optimisations on the generated FOL problem, e.g. replacing some terms of the form *P*(*cts*) with *c*(*t*, *s*).

A FOL *axiom* is a pair of a FOL formula and a constant (label). We translate ${\mathrm {CIC}}_0$ to a set of FOL axioms. The labels are used to indicate which axioms are translations of which lemmas. When we do not mention the label of an axiom, then the label is not important.

### Export of Coq data

The Coq declarations are exported in a straightforward way, translating Coq terms to corresponding terms of ${\mathrm {CIC}}_0$, possibly forgetting some information like e.g. universe constraints on ${\mathrm {Type}}$. We implemented a Coq kernel plugin which exports the Coq kernel data structures. We briefly comment on several aspects of the export.

- Definitions are exported as ${\mathrm {CIC}}_0$ definitions.
- Axioms are exported as ${\mathrm {CIC}}_0$ typing declarations.
- Free variables (e.g. current hypotheses or variables from a currently open section) are exported as ${\mathrm {CIC}}_0$ constants with appropriate typing declarations.
- Inductive types are exported as ${\mathrm {CIC}}_0$ inductive declarations. Induction principles and recursor definitions are exported as separate ${\mathrm {CIC}}_0$ definitions.
- Coinductive types are treated in the same way as inductive types, except that no induction principles or recursor definitions are exported for them.
- Mutual inductive types are exported separately for each constituent inductive type. See Sect. 3.
- The Coq construct cofix is exported to ${\mathtt {fix}}$ in ${\mathrm {CIC}}_0$ with a special flag that affects the evaluation algorithm. We omitted this flag from the description of ${\mathrm {CIC}}_0$ for the sake of simplicity.
- Modules and functors are not exported. Objects inside a module are exported with the name of the module prefixed to the name of the object.
- Universe constraints on ${\mathrm {Type}}$ are not exported. Proofs of paradoxes present in the standard library, e.g., Hurken’s paradox, are explicitly filtered out and not exported.
- The following objects from the Init.Logic module are represented directly by the corresponding logical primitives of ${\mathrm {CIC}}_0$: True, False, all, ex, and, or, iff, eq. No other objects from the Init.Logic module are exported.
- Records are translated to inductive types already by Coq. Primitive record projections are not supported by our plugin.
- Existential metavariables are not exported. Currently it is not possible to use the hammer plugin when the proof state contains some uninstantiated existential metavariables.

The limitations of the translation, including these stemming from the incompleteness of the export as well as of the current architecture will be discussed in Sects. 5.6 and 9.

### Translating Terms

The terms of ${\mathrm {CIC}}_0$ are translated using three mutually recursively defined functions $\mathcal {F}$, $\mathcal {G}$ and $\mathcal {C}$. The function $\mathcal {F}$ encodes propositions as FOL formulas and is used for terms of ${\mathrm {CIC}}_0$ having type ${\mathrm {Prop}}$, i.e., for propositions of ${\mathrm {CIC}}_0$. The function $\mathcal {G}$ encodes types as guards and is used for terms of ${\mathrm {CIC}}_0$ which have type ${\mathrm {Type}}$ but not ${\mathrm {Prop}}$. The function $\mathcal {C}$ encodes ${\mathrm {CIC}}_0$ terms as FOL terms. During the translation we add some fresh constants together with axioms (in FOL) specifying their meaning. Hence, strictly speaking, the codomain of each of the functions $\mathcal {F}$, $\mathcal {G}$ and $\mathcal {C}$ is the Cartesian product of the set of FOL formulas (or terms)—the desired encoding—and the powerset of the set of FOL formulas—the set of axioms added during the translation. However, it is more readable to describe the functions assuming a global mutable collection of FOL axioms.

Our translation assumes proof irrelevance. We use a fresh constant $\mathtt {prf}$ to represent an arbitrary proof object (of any inhabited proposition). For the sake of efficiency, ${\mathrm {CIC}}_0$ propositions are translated directly to FOL formulas using the $\mathcal {F}$ function. The ${\mathrm {CIC}}_0$ types which are not propositions are translated to guards which essentially specify what it means for an object to have the given type. The formula $\mathcal {G}(t, \alpha )$ intuitively means “*t* has type $\alpha$”. For instance, for a (closed) type $\tau = \varPi x : \alpha . \beta$ we have

$$\begin{aligned} \mathcal {G}(f, \tau ) = \forall x . \mathcal {G}(x, \alpha ) \rightarrow \mathcal {G}(f x, \beta ) \end{aligned}$$

So $\mathcal {G}(f,\tau )$ says that an object *f* has type $\tau = \varPi x : \alpha . \beta$ if for any object *x* of type $\alpha$, the application *fx* has type $\beta$ (in which *x* may occur free).

Below we give definitions of the functions $\mathcal {F}$, $\mathcal {G}$ and $\mathcal {C}$. These functions are in fact parameterised by a ${\mathrm {CIC}}_0$ context $\varGamma$, which we write as a subscript. In the description of the functions we implicitly assume that variable names are chosen appropriately so that no unexpected variable capture occurs. Also we assume an implicit global environment *E*. This environment is used for type checking. The typing declarations for ${\mathrm {CIC}}_0$ logical primitives, as described in the previous section, are assumed to be present in *E*. During the translation also some new declarations are added to the environment. We assume all ${\mathrm {CIC}}_0$ constants are also FOL constants, and analogously for variables. We use the notation $t_1 \approx _\varGamma t_2$ for $t_1 \leftrightarrow t_2$ if $\varGamma \vdash t_1 : {\mathrm {Prop}}$, or for $t_1 = t_2$ if $\varGamma \nvdash t_1 : {\mathrm {Prop}}$.

The function $\mathcal {F}$ encoding propositions as FOL formulas:

- If $$\varGamma \vdash t : {\mathrm {Prop}}$$ then $$\mathcal {F}_\varGamma (\varPi x : t . s) =\mathcal {F}_{\varGamma }(t) \rightarrow \mathcal {F}_{\varGamma ,x:t}(s)$$.
- If $$\varGamma \not \vdash t : {\mathrm {Prop}}$$ then $$\mathcal {F}_\varGamma (\varPi x : t . s) = \forall x . \mathcal {G}_{\varGamma }(x, t) \rightarrow \mathcal {F}_{\varGamma ,x:t}(s)$$.
- $$\mathcal {F}_\varGamma (\forall x : t . s) = \forall x . \mathcal {G}_\varGamma (x, t) \rightarrow \mathcal {F}_{\varGamma ,x:t}(s)$$.
- $$\mathcal {F}_\varGamma (\exists x : t . s) = \exists x . \mathcal {G}_\varGamma (x, t) \wedge \mathcal {F}_{\varGamma ,x:t}(s)$$.
- $$\mathcal {F}_\varGamma (t \circ s) = \mathcal {F}_\varGamma (t) \circ \mathcal {F}_\varGamma (s)$$ where $$\circ \in \{\wedge ,\vee ,\leftrightarrow \}$$.
- $$\mathcal {F}_\varGamma (\lnot t) = \lnot \mathcal {F}_\varGamma (t)$$.
- $$\mathcal {F}_\varGamma (t = s) = (\mathcal {C}_\varGamma (t) = \mathcal {C}_\varGamma (s))$$.
- Otherwise, if none of the above apply, $\mathcal {F}_\varGamma (t) = P(\mathcal {C}_\varGamma (t))$.

The function $\mathcal {G}$ encoding types as guards:

- If $w = \varPi x : t . s$ and $\varGamma \vdash t : {\mathrm {Prop}}$ then
  $$\begin{aligned} \mathcal {G}_\varGamma (u, w) = \mathcal {F}_{\varGamma }(t) \rightarrow \mathcal {G}_{\varGamma ,x:t}(u, s). \end{aligned}$$
- If $w = \varPi x : t . s$ and $\varGamma \not \vdash t : {\mathrm {Prop}}$ then $\mathcal {G}_\varGamma (u, w) = \forall x . \mathcal {G}_{\varGamma }(x,t) \rightarrow \mathcal {G}_{\varGamma ,x:t}(u x, s)$.
- If *w* is not a product then $\mathcal {G}_\varGamma (u, w) = T(u, \mathcal {C}_\varGamma (w))$.

The function $\mathcal {C}$ encoding terms as FOL terms:

- $$\mathcal {C}_\varGamma (c) = c$$ for a constant *c*,
- $\mathcal {C}_\varGamma (x) = x$ for a variable *x* if *x* is not a $\varGamma$-proof,
- $\mathcal {C}_\varGamma (x) = \mathtt {prf}$ for a variable *x* if *x* is a $\varGamma$-proof,
- $\mathcal {C}_\varGamma (t s)$ is equal to:
  - $$\mathtt {prf}$$ if $$\mathcal {C}_\varGamma (t) = \mathtt {prf}$$,
  - $$\mathcal {C}_\varGamma (t)$$ if $$\mathcal {C}_\varGamma (t) \ne \mathtt {prf}$$ but $$\mathcal {C}_\varGamma (s) = \mathtt {prf}$$,
  - $$\mathcal {C}_\varGamma (t) \mathcal {C}_\varGamma (s)$$ otherwise.
- $\mathcal {C}_\varGamma (\varPi x : t . s) = R \vec {y}$ for a fresh constant *F* where $\vec {y} = {\mathrm {FF}}_\varGamma ({\mathrm {FC}}(\varGamma ; \varPi x : t . s))$ and
  - if $\varGamma \vdash (\varPi x : t . s) : {\mathrm {Prop}}$ then $\forall \vec {y} . P(F \vec {y}) \leftrightarrow \mathcal {F}_\varGamma (\varPi x : t . s)$ is a new axiom,
  - if $\varGamma \not \vdash (\varPi x : t . s) : {\mathrm {Prop}}$ then $\forall \vec {y} z . T(z, F \vec {y}) \leftrightarrow \mathcal {G}_\varGamma (z, \varPi x : t . s)$ is a new axiom.
- $\mathcal {C}_\varGamma (\lambda \vec {x} : \vec {\tau } . t) = F \vec {y_0}$ for a fresh constant *F* where
  - *t* does not start with a lambda-abstraction any more,
  - $$\varGamma ,\vec {x}:\vec {\tau } \vdash t : \alpha $$,
  - $$\vec {y} : \vec {\rho } = {\mathrm {FC}}(\varGamma ;\lambda \vec {x} : \vec {\tau } . t)$$,
  - $$\vec {y_0} = {\mathrm {FF}}_\varGamma (\vec {y})$$ and $$\vec {x_0} = {\mathrm {FF}}_{\varGamma ,\vec {x}:\vec {\tau }}(\vec {x})$$,
  - the typing declaration $F : \varPi \vec {y} : \vec {\rho } . \varPi \vec {x} : \vec {\tau } . \alpha$ is added to the global environment *E* (before the recursive call to $\mathcal {F}_\varGamma$ below),
  - the following is a new axiom:
    $$\begin{aligned} \forall \vec {y_0} \vec {x_0} . \mathcal {F}_{\varGamma ,\vec {x}:\vec {\tau }}(F \vec {y} \vec {x} \approx _{\varGamma ,\vec {x}:\vec {\tau }} t). \end{aligned}$$
    Note that the call to $\mathcal {F}$ will remove those variable arguments to *F* which are $\varGamma ,\vec {x}:\vec {\tau }$-proofs. Hence, ultimately *F* will occur as $F \vec {y_0} \vec {x_0}$ in the above axiom.
- If *t* is a $\varGamma$-proof then
  $$\begin{aligned} \mathcal {C}_\varGamma ({\mathtt {case}}(t, c, n, \lambda \vec {a} : \vec {\alpha } . \lambda x : c \vec {p} \vec {a}. \tau , \lambda \vec {x_1} : \vec {\tau _1} . s_1, \ldots , \lambda \vec {x_k} : \vec {\tau _k} . s_k)) = C \end{aligned}$$
  for a fresh constant *C*.
- If *t* is not a $\varGamma$-proof then
  $$\begin{aligned} \mathcal {C}_\varGamma ({\mathtt {case}}(t, c, n, \lambda \vec {a} : \vec {\alpha } . \lambda x : c \vec {p} \vec {a}. \tau , \lambda \vec {x_1} : \vec {\tau _1} . s_1, \ldots , \lambda \vec {x_k} : \vec {\tau _k} . s_k)) = F \vec {y_0} \end{aligned}$$
  for a fresh constant *F* where
  - $$I(c : \gamma {:}{=} c_1 : \gamma _1, \ldots , c_k : \gamma _k) \in E$$,
  - $$\vec {y} : \vec {\rho } = {\mathrm {FC}}(\varGamma ; {\mathtt {case}}(t, c, n, \lambda \vec {a} : \vec {\alpha } . \lambda x : c \vec {p} \vec {a}. \tau , \lambda \vec {x_1} : \vec {\tau _1} . s_1, \ldots , \lambda \vec {x_k} : \vec {\tau _k} . s_k))$$,
  - $$\vec {y_0} = {\mathrm {FF}}_{\varGamma }(\vec {y})$$,
  - $$\vec {y_1} : \vec {\rho _1} = {\mathrm {FC}}(\varGamma ;t)$$,
  - $\varGamma \vdash t : c \vec {p} \vec {u}$ for some terms $\vec {u}$,
  - the declaration $F : \varPi \vec {y} : \vec {\rho } . \tau [\vec {u}/\vec {a},t/x]$ is added to the global environment *E*,
  - the following is a new axiom:
    $$\begin{aligned} \begin{array}{rcl} \forall \vec {y_0} . {\mathrm {guards}}_{\vec {y_1} : \vec {\rho _1}}(\mathcal {F}_\varGamma &{}((&{} \exists \vec {x_1} : \vec {\tau _1} . t = c_1 \vec {p} \vec {x_1} \wedge F \vec {y} \approx _{\varGamma ,\vec {x_1}:\vec {\tau _1}} s_1) \\ &{}\vee &{} \ldots \\ &{}\vee &{} (\exists \vec {x_k} : \vec {\tau _k} . t = c_k \vec {p} \vec {x_k} \wedge F \vec {y} \approx _{\varGamma ,\vec {x_k}:\vec {\tau _k}} s_k))) \end{array} \end{aligned}$$
    where for a FOL formula $\varphi$ and a context $\varGamma$ we define ${\mathrm {guards}}_\varGamma (\varphi )$ inductively as follows:
    - $${\mathrm {guards}}_{\langle \rangle }(\varphi ) = \varphi $$,
    - $${\mathrm {guards}}_{\varGamma ,x:\tau }(\varphi ) = {\mathrm {guards}}_\varGamma (\mathcal {F}_\varGamma (\tau ) \rightarrow \varphi )$$ if $$\varGamma \vdash \tau : {\mathrm {Prop}}$$,
    - $${\mathrm {guards}}_{\varGamma ,x:\tau }(\varphi ) = {\mathrm {guards}}_\varGamma (\mathcal {G}_\varGamma (x, \tau ) \rightarrow \varphi )$$ if $$\varGamma \nvdash \tau : {\mathrm {Prop}}$$.
- $$\mathcal {C}_\varGamma ({\mathtt {fix}}(f_j, f_1 : \tau _1 {:}{=} t_1, \ldots , f_n : \tau _n {:}{=} t_n)) = F_j \vec {y_0}$$ where
  - $$\vec {y} : \vec {\alpha } = {\mathrm {FC}}(\varGamma ;{\mathtt {fix}}(f_j, f_1 : \tau _1 {:}{=} t_1, \ldots , f_n : \tau _n {:}{=} t_n))$$,
  - $$\vec {y_0} = {\mathrm {FF}}_\varGamma (\vec {y})$$,
  - $F_1,\ldots ,F_n$ are fresh constants,
  - for $i=1,\ldots ,n$ the typing declarations $F_i : \varPi \vec {y} : \vec {\alpha } . \tau _i$ are added to the global environment *E*,
  - for $i=1,\ldots ,n$ the following are new axioms:
    $$\begin{aligned} \forall \vec {y_0} . {\mathcal {F}}_\varGamma (F_i \vec {y} \approx _\varGamma t_i[F_1 \vec {y}/f_1,\ldots ,F_n \vec {y}/f_n]). \end{aligned}$$
- $\mathcal {C}_\varGamma ({\mathtt {let}}(x : \tau {:}{=} t, s)) = \mathcal {C}_\varGamma (s[F\vec {y_0}/x])$ for a fresh constant *F* where
  - $$\vec {y} : \vec {\alpha } = {\mathrm {FC}}(\varGamma ;t \tau )$$,
  - $$\vec {y_0} = {\mathrm {FF}}_\varGamma (\vec {y})$$,
  - $$\sigma = \varPi \vec {y} : \vec {\alpha } . \tau $$,
  - the definition $F = (\lambda \vec {y} : \vec {\alpha } . t) : \sigma$ is added to the global environment *E* (before the recursive call to $\mathcal {C}_\varGamma$ above),
  - if $\nvdash \sigma : {\mathrm {Prop}}$ then $\forall \vec {y_0} . F \vec {y_0} = \mathcal {C}_\varGamma (t)$ is a new axiom.
- $$\mathcal {C}_\varGamma ({\mathtt {cast}}(\mathtt {prf},\tau )) = \mathtt {prf}$$.
- If $t \ne \mathtt {prf}$ then $\mathcal {C}_\varGamma ({\mathtt {cast}}(t,\tau )) = F \vec {y_0}$ for a fresh constant *F* where
  - $$\vec {y} : \vec {\alpha } = {\mathrm {FC}}(\varGamma ; t \tau )$$,
  - $$\vec {y_0} = {\mathrm {FF}}_\varGamma (\vec {y})$$,
  - $$\sigma = \varPi \vec {y} : \vec {\alpha } . \tau $$,
  - the definition $F = (\lambda \vec {y} : \vec {\alpha } . t) : \sigma$ is added to the global environment *E*,
  - if $\nvdash \sigma : {\mathrm {Prop}}$ then $\forall \vec {y_0} . F \vec {y_0} = \mathcal {C}_\varGamma (t)$ is a new axiom.

#### Example 1

A $${\mathrm {CIC}}_0$$ proposition

$$\begin{aligned} t = \varPi x : N . \varPi f : \alpha \rightarrow N \rightarrow N . \varPi q : \alpha . f q x = x \end{aligned}$$

in the context

$$\begin{aligned} \varGamma = N : {\mathrm {Type}}, \alpha : Prop \end{aligned}$$

is translated to

$$\begin{aligned} {\mathcal {F}}_\varGamma (t) = \forall x . T(x, N) \rightarrow \forall f . (P(\alpha ) \rightarrow \forall y . T(y, N) \rightarrow T(f y, N)) \rightarrow P(\alpha ) \rightarrow f x = x. \end{aligned}$$

In practice, checking the conditions $\varGamma \vdash t : {\mathrm {Prop}}$ is performed by our specialised approximate proposition-checking algorithm. Checking whether a term *t* is a $\varGamma$-proof occurs in two cases.

1. *t* is the term matched on in a ${\mathtt {case}}$-expression ${\mathtt {case}}(t,c,\ldots )$. Then there is an inductive declaration $I_n(c : \gamma {:}{=} \ldots )$ in the global environment. We check if the normal form of $\gamma$ has target ${\mathrm {Prop}}$.
2. $t=x$ is a variable. Then we check if the type assigned to *x* by the context $\varGamma$ is a proposition.

We write $\varphi (\sigma )$ to denote that a FOL formula $\varphi$ has $\sigma$ as a subformula. Then $\varphi (\sigma ')$ denotes the formula $\varphi$ with $\sigma$ replaced by $\sigma '$. We use an analogous notation when $\sigma$ is a FOL term instead of a formula.

Note that each new axiom defining a constant *F* intended to replace (“lift-out”) a $\lambda$-abstraction, a case expression or a fixpoint definition has the form

$$\begin{aligned} \forall \vec {x} . \varphi (F \vec {x} = t) \end{aligned}$$

or

$$\begin{aligned} \forall \vec {x} . \varphi (P(F \vec {x}) \leftrightarrow \psi ). \end{aligned}$$

We will call each such axiom the *lifting axiom for F*. For lambda abstractions, this is equivalent to lambda-lifing, which is a common technique used by hammers for HOL and Mizar. In ${\mathrm {CIC}}_0$ however other kinds of terms do bind variables (for example case and fix) and lifting axioms need to be created for such terms as well.

### Translating Declarations

Declarations of ${\mathrm {CIC}}_0$ are encoded as FOL axioms. As before, a global ${\mathrm {CIC}}_0$ environment *E* is assumed. During the translation of a declaration the functions $\mathcal {F}$, $\mathcal {G}$ and $\mathcal {C}$ from the previous subsection are used. These functions may themselves add some FOL axioms, which are then also included in the result of the translation of the declaration. We proceed to describe the translation for each of the three forms of ${\mathrm {CIC}}_0$ declarations. Whenever we write $\mathcal {F}$, $\mathcal {G}$, $\mathcal {C}$ without subscript, the empty context $\langle \rangle$ is assumed as the subscript.

A definition $c = t : \tau$ is translated as follows.

- If $\vdash \tau : {\mathrm {Prop}}$ then add $\mathcal {F}(\tau )$ as a new axiom with label *c*.
- If $$\nvdash \tau : {\mathrm {Prop}}$$ then
  - add $\mathcal {G}(c, \tau )$ as a new axiom,
  - if $\tau = {\mathrm {Prop}}$ then add $c \leftrightarrow \mathcal {F}(t)$ as a new axiom with label *c*,
  - if $\tau = {\mathrm {Set}}$ or $\tau = {\mathrm {Type}}$ then add $\forall f . c f \leftrightarrow \mathcal {G}(f, t)$ as a new axiom with label *c*,
  - if $\tau \notin \{{\mathrm {Prop}},{\mathrm {Set}},{\mathrm {Type}}\}$ then add $c = \mathcal {C}(t)$ as a new axiom with label *c*.

A typing declaration $c : \tau$ is translated as follows.

- If $\vdash \tau : {\mathrm {Prop}}$ then add $\mathcal {F}(\tau )$ as a new axiom with label *c*.
- If $\nvdash \tau : {\mathrm {Prop}}$ then add $\mathcal {G}(c, \tau )$ as a new axiom with label *c*.

An inductive declaration $I(c : \tau {:}{=} c_1 : \tau _1, \ldots , c_n : \tau _n)$ is translated as follows, where $\tau \Downarrow \varPi \vec {p} : \vec {\beta } . \varPi \vec {y} : \vec {\gamma } . s$ and $s \in \{{\mathrm {Prop}},{\mathrm {Set}},{\mathrm {Type}}\}$ and $\vec {\beta }$ are the types of the parameters of the inductive type and $\tau _i \Downarrow \varPi \vec {p} : \vec {\beta } . \varPi \vec {x_i} : \vec {\alpha _i}. c \vec {p} \vec {t_i}$ and the length of $\vec {y}$ and each $\vec {t_i}$ is *m*.

- Translate the typing declaration $c : \tau$.
- Translate each typing declaration $c_i : \tau _i$ for $i=1,\ldots ,n$.
- If $s \ne {\mathrm {Prop}}$ then for each $i=1,\ldots ,n$ add the following injectivity axiom:
  $$\begin{aligned} \mathcal {F}(\forall \vec {x_i} : \vec {\alpha _i} . \forall \vec {x_i}' : \vec {\alpha _i}' . c_i \vec {x_i} = c_i \vec {x_i}' \rightarrow x_{i,1} = x_{i,1}' \wedge \ldots \wedge x_{i,k_i} = x_{i,k_i}') \end{aligned}$$
  where $$\vec {\alpha _i}' = \vec {\alpha _i}[\vec {x_i}'/\vec {x_i}]$$.
- If $s \ne {\mathrm {Prop}}$ then for each $i,j=1,\ldots ,n$ with $i \ne j$ add the following discrimination axiom:
  $$\begin{aligned} \mathcal {F}(\forall \vec {x_i} : \vec {\alpha _i} . \forall \vec {x_j} : \vec {\alpha _j} . c_i \vec {x_i} \ne c_j \vec {x_j}). \end{aligned}$$
- If $s \ne {\mathrm {Prop}}$ then add the following inversion axiom:
  $$\begin{aligned} \begin{array}{rcl} \mathcal {F}(\forall \vec {p} : \vec {\beta } . \forall \vec {y} : \vec {\gamma } . \forall z : c \vec {p} \vec {y} &{}.&{} (\exists \vec {x_1} : \vec {\alpha _1} . z = c_1 \vec {p} \vec {x_1} \wedge y_1 = t_{1,1} \wedge \ldots \wedge y_m = t_{1,m}) \\ &{}\vee &{} \ldots \\ &{}\vee &{} (\exists \vec {x_n} : \vec {\alpha _n} . z = c_n \vec {p} \vec {x_n} \wedge y_1 = t_{n,1} \wedge \ldots \wedge y_m = t_{n,m})). \end{array} \end{aligned}$$
- If $s = {\mathrm {Prop}}$ then add the following inversion axiom:
  $$\begin{aligned} \begin{array}{rcl} \mathcal {F}(\forall \vec {p} : \vec {\beta } . \forall \vec {y} : \vec {\gamma } . c \vec {p} \vec {y} \rightarrow &{}((&{}\exists \vec {x_1} : \vec {\alpha _1} . y_1 = t_{1,1} \wedge \ldots \wedge y_m = t_{1,m}) \\ &{}\vee &{} \ldots \\ &{}\vee &{} (\exists \vec {x_n} : \vec {\alpha _n} . y_1 = t_{n,1} \wedge \ldots \wedge y_m = t_{n,m}))). \end{array} \end{aligned}$$

### Translating Problems

A ${\mathrm {CIC}}_0$ problem consists of a set of assumptions which are ${\mathrm {CIC}}_0$ declarations, and a conjecture which is a ${\mathrm {CIC}}_0$ proposition. A ${\mathrm {CIC}}_0$ problem is translated to a FOL problem by translating the assumptions to FOL axioms in the way described in the previous subsection, and translating the conjecture *t* to a FOL conjecture $\mathcal {F}(t)$. New declarations added to the environment during the translation are *not* translated. For every ${\mathrm {CIC}}_0$ problem the following FOL axioms are added to the result of the translation:

- $$T({\mathrm {Prop}},{\mathrm {Type}})$$, $$T({\mathrm {Set}},{\mathrm {Type}})$$, $$T({\mathrm {Type}},{\mathrm {Type}})$$,
- $$\forall y . T(y,{\mathrm {Set}}) \rightarrow T(y,{\mathrm {Type}})$$.

### Optimisations

We perform the following optimisations on the generated FOL problems, in the given order. Below, by an occurrence of a term *t* (in the FOL problem) we mean an occurrence of *t* in the set of FOL formulas comprising the given FOL problem.

- We recursively simplify the lifting axioms for the constants encoding $\lambda$-abstractions, case expressions and fixpoint definitions. For any lifting axiom *A* for a constant *F*, if *A* has the form
  $$\begin{aligned} \forall \vec {x} . \varphi (F \vec {x} = G \vec {x}) \end{aligned}$$
  such that *G* has a lifting axiom *B*
  $$\begin{aligned} \forall \vec {x} \forall \vec {y} . \psi (G \vec {x} \vec {y} = t) \end{aligned}$$
  and either $\varphi (\Box ) = \Box$ or $\vec {y}$ is empty, then we replace the axiom *A* by
  $$\begin{aligned} \forall \vec {x} . \varphi (\forall \vec {y} . \psi (F \vec {x} \vec {y} = t)) \end{aligned}$$
  and we remove the axiom *B* and replace all occurrences of *G* by *F*. When in the lifting axioms *A* and *B* we have logical equivalence $\leftrightarrow$ instead of equality $=$, then we adjust the replacement of *A* appropriately, using $\leftrightarrow$ instead of $=$. We repeat applying this optimisation as long as possible.
- For a constant *c*, we replace any occurrence of $T(s, c t_1 \ldots t_n)$ by $c_T(t_1,\ldots ,t_n,s)$ where $c_T$ is a new function symbol of arity $n+1$. We then also add a new axiom:
  $$\begin{aligned} \forall x_1 \ldots x_n y . c_T(x_1,\ldots ,x_n,y) \leftrightarrow T(y, c x_1 \ldots x_n). \end{aligned}$$
  Note that after performing this replacement the predicate *T* may still occur in the FOL problem, e.g., a term $T(s, x t_1 \ldots t_n)$ may occur. This optimisation is useful, because it simplifies the FOL terms and replaces the *T* predicate with a specialised predicate for a constant. This makes it easier for the ATPs to handle the problem.
- For each occurrence of a constant *c* with $n > 0$ arguments, i.e., each occurrence $c t_1 \ldots t_n$ where $n > 0$ is maximal (there are no further arguments), we replace this occurrence with $c^n(t_1,\ldots ,t_n)$ where $c^n$ is a new *n*-ary function symbol. We then also add a new axiom:
  - $\forall x_1 \ldots x_n . P(c^n(x_1,\ldots ,x_n)) \leftrightarrow P(c x_1 \ldots x_n)$ if (after replacement of all such occurrences) all terms of the form $c^n(t_1,\ldots ,t_n)$ occur only as arguments of the predicate *P*, i.e., occur only as in $P(c^n(t_1,\ldots ,t_n))$.
  - $$\forall x_1 \ldots x_n . c^n(x_1,\ldots ,x_n) = c x_1 \ldots x_n$$ otherwise.
  This optimisation is similar to the optimisation originally described by Meng and Paulson in [61, Section 2.7].
- For any constant *c* and $n>0$, if all terms of the form $c^n(t_1,\ldots ,t_n)$ occur only as arguments of *P*, then replace each occurrence of a term of the form $P(c^n(t_1,\ldots ,t_n))$ by $c^n(t_1,\ldots ,t_n)$.

### Properties of the Translation

In this section we briefly comment on the theoretical aspects of the translation. Further limitations of the whole approach will be mentioned in Sect. 9. The translation is neither sound nor complete. The lack of soundness is caused e.g. by the fact that we forget universe constraints on ${\mathrm {Type}}$, the assumption of proof irrelevance, and the combination of omitting type guards for lifted-out lambda-abstractions with translating Coq equality to FOL equality. However, our experimental evaluation indicates that the translation is both sound and complete “enough” to be practically usable. Also, a “core” version of our translation is sound. A soundness proof and a more detailed discussion of the theoretical properties of a core version of our translation may be found in [27].

Note that e.g. in the axiom added for lifted-out lambda-abstractions

$$\begin{aligned} \forall \vec {y_0} \vec {x_0} . \mathcal {F}_{\varGamma ,\vec {x}:\vec {\tau }}(F \vec {y} \vec {x} \approx _{\varGamma ,\vec {x}:\vec {\tau }} t) \end{aligned}$$

we do not generate type guards for the free ($\vec {y_0}$) or bound ($\vec {x_0}$) variables of the lambda-expression. In practice, omitting these guards slightly improves the success rate of the ATPs without significantly affecting the reconstruction success rate. We conjecture that, ignoring other unsound features of the translation, omitting these guards is sound provided that the inductive Coq equality type eq is *not* translated to FOL equality. Note also that it is not sound (and our translation does not do it) to omit guards for the free variables of the term matched on in the ${\mathtt {case}}$ construct, even if Coq equality is not translated to FOL equality. For example, assume $I_0(c : {\mathrm {Set}}{:}{=} c_0 : c)$ is in the global environment. With the guards omitted, for the ${\mathtt {case}}$-expression ${\mathtt {case}}(x,c,0,c,c_0)$ we would add an axiom

$$\begin{aligned} \forall x . x = c_0 \wedge F x = c_0 \end{aligned}$$

with *F* a fresh first-order constant. This obviously leads to an inconsistency by substituting for *x* two distinct constants $c_1,c_2$ such that $c_1 \ne c_2$ is provable.

In our translation we map Coq equality to FOL equality which is not sound in combination with omitting the guards for free variables. In particular, if a ${\mathrm {CIC}}_0$ problem contains a functional extensionality axiom then the generated FOL problem may be inconsistent, and in contrast to the inconsistencies that may result from omitting certain universe constraints, this inconsistency may be “easy enough” for the ATPs to derive. Our plugin has an option to turn on guard generation for free variables. See also [27, Section 6].

## Proof Reconstruction

In this section we will discuss a number of existing Coq internal automation mechanisms that could be useful for proof reconstruction and finally introduce our combined proof reconstruction tactic.

The tactic firstorder is based on an extension of the contraction-free sequent calculus LJT of Dyckhoff [32] to first-order intuitionistic logic with inductive definitions [26]. A decision procedure for intuitionistic propositional logic based on the system LJT is implemented in the tactic tauto. The tactic firstorder does not take into account many features of Coq outside of first-order logic. In particular, it does not fully axiomatise equality.

In general, the tactics based on extensions of LJT do mostly forward reasoning, i.e., they predominantly manipulate the hypotheses in the context to finally obtain the goal. Our approach is based more on an auto-type proof search which does mostly backward Prolog-style reasoning—modifying the goal by applying hypotheses from the context. The core of our search procedure may be seen as an extension of the Ben-Yelles algorithm [21, 42] to first-order intuitionistic logic with all connectives [71, 75]. It is closely related to searching for $\eta$-long normal forms [12, 31]. Our implementation extends this core idea with various heuristics. We augment the proof search procedure with the use of existential metavariables like in eauto, a looping check, some limited forward reasoning, the use of the congruence tactic, and heuristic rewriting using equational hypotheses.

It is important to note that while the external ATPs we employ are classical and the translation assumes proof irrelevance, the proof reconstruction phase does not assume any additional axioms. We re-prove the theorems in the intuitionistic logic of Coq, effectively using the output of the ATPs merely as hints for our hand-crafted proof search procedure. Therefore, if the ATP proof is inherently classical then proof reconstruction will fail. Currently, the only information from ATP runs we use is a list of lemmas needed by the ATP to prove the theorem (these are added to the context) and a list of constant definitions used in the ATP proof (we try unfolding these constants and no others).

Another thing to note is that we do not use the information contained in the Coq standard library during reconstruction. This would not make sense for our evaluation of the reconstruction mechanism, since we try to re-prove the theorems from the Coq standard library. In particular, we do not use any preexisting hint databases available in Coq, not even the core database (for the evaluation we use the auto and eauto tactics with the nocore option, but in the final version of the reconstruction tactics we also use auto without this option). Also, we do not use any domain-specific decision procedures available as Coq tactics, e.g., field, ring or omega. Including such techniques in HOLyHammer did allow fast solving of many simple arithmetic problems [53].

We now describe a simplification of our proof search procedure. We will treat the current proof state as a collection of judgements of the form $\varGamma \vdash G$ and describe the rules as manipulating a single such judgement. In a judgement $\varGamma \vdash G$ the term *G* is the *goal* and $\varGamma$ is the *context* which is a list of *hypothesis* declarations of the form *H* : *A*. We use an informal notation for Coq terms similar to how they are displayed by Coq. For instance, by $\forall x : A, B$ we denote a dependent product. We write $\forall x, B$ when the type of *x* is not essential. Note that in $\forall x, B$ the variable *x* may be a proposition, so $\forall x, B$ may actually represent a logical implication $A \rightarrow B$ if *A* is the omitted type of *x* which itself has type ${\mathrm {Prop}}$ and *x* does not occur in *B*. To avoid confusion with $=$ used to denote the equality inductive predicate in Coq, we use $\equiv$ as a metalevel symbol to denote identity of Coq terms. We use the notation $\varGamma ; H : A$ to denote $\varGamma$ with *H* : *A* inserted at some fixed position. By $\varGamma , H : A$ we denote the context $\varGamma$ with *H* : *A* appended. We omit the hypothesis name *H* when irrelevant. By *C*[*t*] we denote an occurrence of a term *t* in a term context *C*.

**Fig. 1:**
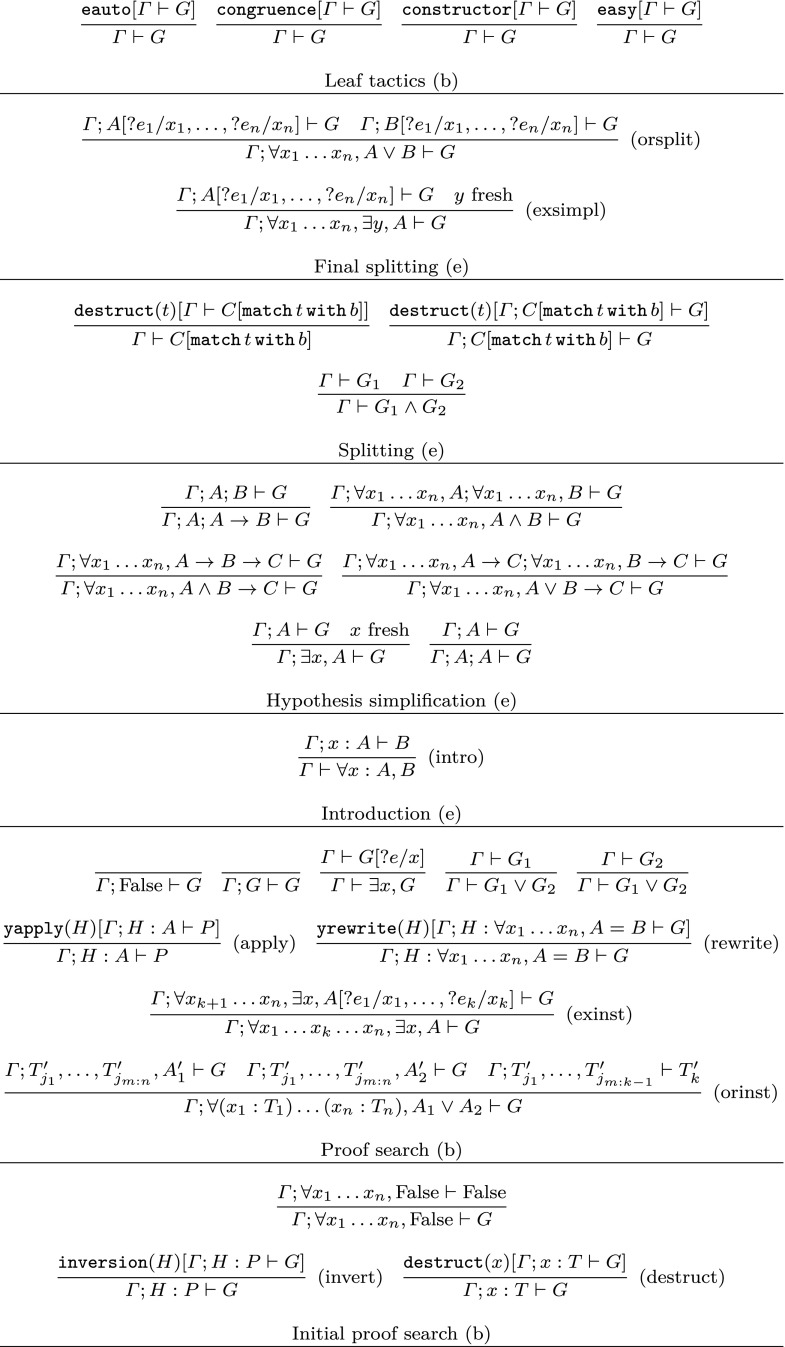
Simplified proof search rules

The proof search procedure applies the rules from Fig. 1. An application of a rule of the form

$$\begin{aligned} \frac{\varGamma _1 \vdash G_1 \;\;\; \ldots \;\;\; \varGamma _n \vdash G_n}{\varGamma \vdash G} \end{aligned}$$

replaces a judgement $\varGamma \vdash G$ in the current proof state by the judgements $\varGamma _1 \vdash G_1$, ..., $\varGamma _n \vdash G_n$. The notation ${\texttt {tac}}[\varGamma \vdash G]$ (resp. ${\texttt {tac}}(A)[\varGamma \vdash G]$) in a rule premise means applying the Coq tactic tac (with argument *A*) to the judgement $\varGamma \vdash G$ and making the judgements (subgoals) generated by the tactic be the premises of the rule. In a rule of the form e.g.

$$\begin{aligned} \frac{\varGamma ; A' \vdash G}{\varGamma ; A \vdash G} \end{aligned}$$

the position in $\varGamma$ at which *A* is inserted is implicitly assumed to be the same as the position at which $A'$ is inserted.

In Fig. 1 the variables $?e_i$, ?*e* denote fresh existential metavariables of appropriate types. These metavariables need to be instantiated later by Coq’s unification algorithm. In the rules $({\mathrm {orsplit}})$ and $({\mathrm {exsimpl}})$ the types of $x_1,\ldots ,x_n$ are assumed not to be propositions. In the rule $({\mathrm {exinst}})$ the types of $x_1,\ldots ,x_k$ are not propositions and either $k = n$ or the type of $x_{k+1}$ is a proposition. In the rule $({\mathrm {orinst}})$ the $x_{i_1},\ldots ,x_{i_m}$ are all those among $x_1,\ldots ,x_n$ for which $T_{i_1},\ldots ,T_{i_m}$ are not propositions; and the index *k* ranges over all $k \in \{1,\ldots ,n\} \setminus \{i_1,\ldots ,i_m\}$ (so that each $T_k$ is a proposition)—all judgements for any such *k* are premises of the rule, not just a single one. Moreover, in these rules for any term *T* by $T'$ we denote $T[?e_{i_1}/x_{i_1},\ldots ,?e_{i_m}/x_{i_m}]$, and $T_{j_1},\ldots ,T_{j_{m:k}}$ are those among $T_1,\ldots ,T_k$ which are propositions. In the $({\mathrm {apply}})$ and $({\mathrm {invert}})$ rules *P* is an atomic proposition, i.e., a proposition which is not a dependent product, an existential, a disjunction or a conjunction. In the $({\mathrm {destruct}})$ rule *T* is not a proposition.

The tactic yapply in rule $({\mathrm {apply}})$ works like eapply except that instead of simply unifying the goal with the target of the hypothesis, it tries unification modulo some simple equational reasoning. The idea of the yapply tactic is broadly similar to the smart matching of Matita [8], but our implementation is more heuristic and not based on superposition.

The tactic yrewrite in rule $({\mathrm {rewrite}})$ uses Coq’s tactic erewrite to try to rewrite the hypothesis in the goal. If it fails to rewrite it directed from left to right, then it tries the other direction.

The rules in Fig. 1 are divided into groups. The rules in each group are either applied with backtracking (marked by (b) in the figure), i.e., if applying one of the rules in the group to a judgement $\varGamma \vdash G$ does not ultimately succeed in finishing the proof then another of the rules in the group is tried on $\varGamma \vdash G$; or they are applied eagerly without backtracking (marked by (e) in the figure). There are also restrictions on when the rules in a given group may be applied. The rules in the group “Leaf tactics” must close a proof tree branch, i.e., they are applied only when they generate zero premises. The rules in the group “Final splitting” are applied only before the “leaf tactics”. The rules in the groups “Splitting”, “Hypothesis simplification” and “Introduction” are applied whenever possible. The rules in the group “Proof search” constitute the main part of the proof search procedure. They are applied only when none of the rules in the groups “Splitting”, “Hypothesis simplification” and “Introduction” can be applied. The rules in the group “Initial proof search” may only be applied after an application of $({\mathrm {intro}})$ followed by some applications of the rules in the “Splitting” and “Hypothesis simplification” groups. They are applied only if none of the rules in the groups “Splitting”, “Hypothesis simplification” and “Introduction” can be applied.

The above description is only a readable approximation of what is actually implemented. Some further heuristics are used and more complex restrictions are put on what rules may be applied when. In particular, some loop checking (checking whether a judgement repeats) is implemented, the number of times a hypothesis may be used for rewriting is limited, and we also use heuristic rewriting in hypotheses and heuristic instantiation of universal hypotheses. Some heuristics we use are inspired by the crush tactic of Adam Chlipala [23].

As mentioned before, our proof search procedure could be seen as an extension of a search for $\eta$-long normal forms for first-order intuitionistic logic using a Ben-Yelles-type algorithm [71, 75]. As such it would be complete for the fragment of type theory “corresponding to” first-order logic, barring two simplifications we introduced to make it more practical. For the sake of efficiency, we do not backtrack on instantiations of existential metavariables solved by unification, and the rules $({\mathrm {exinst}})$ and $({\mathrm {orinst}})$ are not general enough. These cause incompleteness even for the first-order fragment, but this incompleteness does not seem to matter much in practice. The usual reasons why proof reconstruction fails is that either the proof is inherently classical, too deep, or uses too much rewriting which cannot be easily handled by our rewriting heuristics. It is left for future work to integrate rewriting into our proof search procedure in a more principled way.

The proof reconstruction phase in the hammer tactic uses a number of tactics derived from the procedure described above, with different depth limits, a bit different heuristics and rule application restrictions; plus a few other tactics, including Coq’s intuition, simpl, subst, and heuristic constant unfolding. Various reconstruction tactics are tried in order with a time limit for each, until one of them succeeds (or none succeed—then the proof cannot be reconstructed).

It is important to note that no time limits are supposed to be present in the final proof scripts. The CoqHammer plugin shows which of the tactics succeeded, and the user is supposed to copy this tactic, replacing the hammer tactic invocation. The final reconstruction tactic does not rely on any time limits or make any calls to external ATPs. Its results are therefore completely reproducible on different machines, in contrast to the main hammer tactic itself.

## Integrated Hammer and Evaluation

In this section we present the technique used to select the combination of strategies included in the integrated hammer and present an evaluation of the components as well as the final offered strategy.

The evaluation in this section will perform a push-button re-proving of Coq problems without using their proofs. In order for the evaluation of the system to be fair, we need ensure that no information from a proof is used in its re-proving, as well as that the actual strategy that is used by the whole system has been developed without the knowledge of the proofs being evaluated.

The system will be evaluated on the problems generated from all theorems in the Coq standard library of Coq version 8.5 (a version of the plugin works with Coq 8.6 and 8.7 as well). The problems were generated from the source code of the library, counting as theorems all definitions (introduced with any of Lemma, Theorem, Corollary, Fact, Instance, etc.) that were followed by the Proof keyword. The source code of the library was then modified to insert a hook to our hammer plugin after each Proof keyword. The plugin tries to re-prove the theorem using the Coq theorems accessible at the point when the statement of the theorem is introduced, using the three phases of premise selection, ATP invocation and proof reconstruction as described above.

This simulates how a hammer would be used in the development of the Coq standard library. In particular, when trying to re-prove a given theorem we use only the objects accessible in the Coq kernel at the moment the theorem statement is encountered by Coq. Of course, neither the re-proved theorem itself nor any theorems or definitions that depend on it are used. The number of problems obtained by automatically analysing the Coq standard library source code in the way described above is 9276. This differs significantly from the number of problems reported in [24]. There the theorems in the Coq standard library were extracted from objects of type ${\mathrm {Prop}}$ in the Coq kernel. Because of how the Coq module system works, there may be many Coq kernel objects corresponding to one definition in a source file (this is the case e.g. when using the Include command).

Furthermore, the problems are divided in a training set consisting of about 10% of the problems in the standard library and a validation set containing the remaining 90% of the problems. The training set is used to find a set of complementary strategies. Just like for the hammers for higher-order logic based systems and for Mizar a single best combination of the premise-selection algorithm, number of selected premises, and ATP run for a longer time is much weaker than running a few such combinations even for a shorter time. Contrary to existing hammer constructions [52, 55], we decided to include the reconstruction mechanism among the considered strategy parameters since generally reconstruction rates are lower and it could happen that proofs originating from a particular prover and number of premises would be too hard to reconstruct.

In our evaluation we used the following ATPs: E Prover version 1.9 [65], Vampire version 4.0 [57] and Z3 version 4.0 [28]. The evaluation was performed on a 48-core server with 2.2GHz AMD Opteron CPUs and 320GB RAM. Each problem was always assigned one CPU core. The two considered premise selection algorithms were asked for an ordering of premises, and all powers of two between 16 and 1024 were considered. Finally we considered both firstorder and hrecon reconstruction. Having evaluated all combinations of premise selection algorithms we ordered them in a greedy sequence: each following strategy is the one that adds most to the current selection of strategies. The first 14 strategies in the greedy sequence are presented in Table 1. The column “Solved” indicates the number of problems that were successfully solved by the given ATP with the given premise selection method and a given number of premises, *and* they could be reconstructed by the proof reconstruction procedure described in Sect. 6. The ATPs were run with a time limit of 30 s. The maximum time limit for a single reconstruction tactic was 10 s, depending on the tactic, as described in Sect. 6. No time limit was placed on the premise selection phase, however for goals with largest number of available premises the time does not exceed 0.5 s for either of the considered algorithms. The first strategy that includes firstorder appears only on twelfth position in the greedy sequence and is therefore not used as part of the hammer. We show cumulative success rates to display the progress in the greedy sequence.

The results of the hammer strategies including the premise selection are very good in comparison with the results on the dependencies. Evaluating the translation with hrecon reconstruction is presented in Table 2. The results are significantly worse, mainly for two reasons. First, some dependencies are missing due to our way of recording them which does not take into account the delta-conversion. Secondly, the dependencies in proof terms often were added by automated tactics and are difficult to use for the ATPs. It is sometimes easier for the ATPs to actually prove the theorem from other lemmas in the library than from the original dependencies.

**Table 1:** Success rates of the strategies on the training set in the greedy sequence order

Prover	Selection	Premises	Reconstruction	Solved (%)	Solved
Vampire	k-NN	1024	Hrecon	30.778	285
Z3	k-NN	128	Hrecon	37.473	347
E-Prover	k-NN	1024	Hrecon	39.741	368
Vampire	k-NN	64	Hrecon	40.929	379
Z3	n. Bayes	32	Hrecon	41.469	384
Z3	n. Bayes	512	Hrecon	42.009	389
Z3	n. Bayes	128	Hrecon	42.549	394
E-Prover	n. Bayes	256	Hrecon	43.089	399
Z3	n. Bayes	16	Hrecon	43.521	403
E-Prover	n. Bayes	1024	Hrecon	43.952	407
Vampire	n. Bayes	256	Hrecon	44.276	410
Z3	k-NN	64	Hrecon	44.492	412
Vampire	k-NN	512	Hrecon	44.708	414
E-Prover	k-NN	512	Firstorder	44.924	416
total				46.112	427

**Table 2:** Prover results on the dependencies

Prover	Solved (%)	Solved
Vampire	24.749	2292
Z3	23.961	2219
E-Prover	23.162	2145
Total	26.747	2477

Given the common hardware configuration of computers today, we consider as the integrated system a combination of eight complementary strategies. The final results of the hammer including reconstruction on the validation set are presented in Table 3.

**Table 3:** The success rate of of the combination of strategies on the validation set

Prover	Selection	Premises	Reconstruction	Solved (%)	Solved
Vampire	k-NN	1024	Hrecon	28.816	2673
E-Prover	k-NN	1024	Hrecon	25.593	2374
Vampire	k-NN	64	Hrecon	25.367	2353
Z3	n. Bayes	128	Hrecon	24.299	2254
Z3	k-NN	128	Hrecon	24.127	2238
Z3	n. Bayes	512	Hrecon	23.243	2156
Z3	n. Bayes	32	Hrecon	19.028	1765
E-Prover	n. Bayes	256	Hrecon	17.497	1623
Total				40.815	3786

## Case Studies

The intended use of a hammer is to prove relatively simple goals using available lemmas. The main problem a hammer system tries to solve is that of finding appropriate lemmas in a large collection and combining them to prove the goal. The advantage of a hammer over specialised domain-specific tactics is that it is a general system not depending on any domain knowledge. The hammer plugin may use all currently accessible lemmas, which includes lemmas proven earlier in a given formalization, not only the lemmas from the standard library or other predefined libraries.

It sometimes happens that the ATPs find proofs with fewer dependencies than the proofs in the standard library. One example is the Coq lemma isometric_rotation:
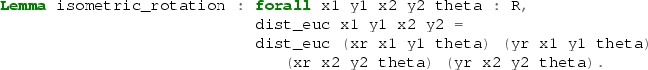


Its current proof in the Coq standard library uses 6 auxiliary facts and is performed using the following 7 line script: 
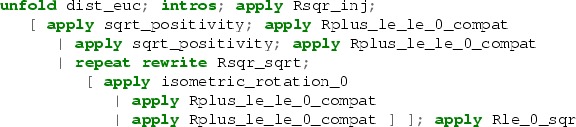


Multiple ATPs found a shorter proof which uses only two of the dependencies: the definition of euclidean distance and the lemma isometric_rotation_0. This suggests that the proof using the injectivity of square root is a detour, and indeed it is possible to write a much simpler valid Coq proof of the lemma using just the two facts used by the ATPs: 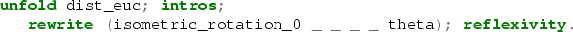


The proof may also be reconstructed from the found dependencies inside Coq. This is also the case for all other examples presented in this section.

Also for some theorems the ATPs found proofs which use premises not present in the dependencies extracted from the proof of the theorems in the standard library. An example is the lemma le_double from Reals.ArithProp: 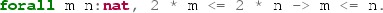 The proof of this lemma in the standard library uses 6 auxiliary lemmas and is performed by the following proof script (two lemmas not visible in the script were added by the tactic prove_sup0): 
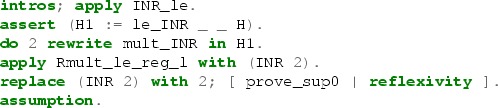
 ATPs found a proof of le_double using only 3 lemmas: Arith.PeanoNat.Nat.le_0 _l, Arith.Mult.mult_S_le_reg_l and Init.Peano.le_n. None of these lemmas appear among the original dependencies.

Another example of hammer usage is a proof of the following fact: 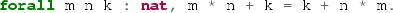 This cannot be proven using the omega tactic because of the presence of multiplication. The tactic invocations eauto with arith or firstorder with arith do not work either. The hammer tool finds a proof using two lemmas from Arith.PeanoNat.Nat: add_comm and mul_comm.

A similar example is the goal 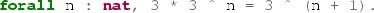 This goal cannot be solved using standard Coq tactics, including the tactic omega. Z3 with 128 preselected premises found a proof using the following lemmas from Arith.PeanoNat.Nat: add_succ_r, le_0_l, pow_succ_r, add_0_r. The proof may be reconstructed using hexhaustive 0 or hyelles 5 tactic invocations.

The next example of a goal solvable by the hammer involves operations on lists. 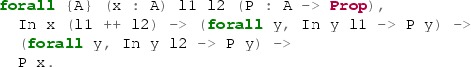 This goal cannot be solved (in reasonable time) using either eauto with datatypes or firstorder with datatypes. The hammer solves this goal using just one lemma: Lists.List.in_app_iff.

A similar example is
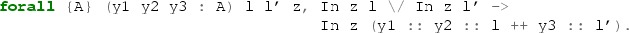
This goal cannot be solved using standard Coq tactics. Eprover with 512 preselected premises found a proof using two lemmas from Lists.List: in_cons and in_or_app.

The hammer is currently not capable of reasoning by induction, except in some very simple cases. Here is an example of a goal where induction is needed. 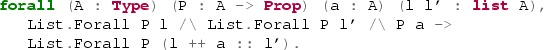 This goal can be solved neither by standard Coq tactics nor by the hammer. However, it suffices to issue the ltac command induction l and the hammer can solve the resulting two subgoals, none of which could be solved by standard Coq tactics. The subgoal for induction base is: 
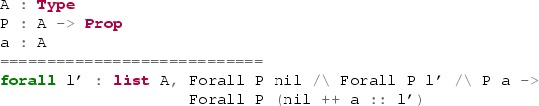
 The hammer solves this goal using the lemma Forall_cons from Lists.List and the definition of ++ (Datatypes.app). The subgoal for the induction step is:
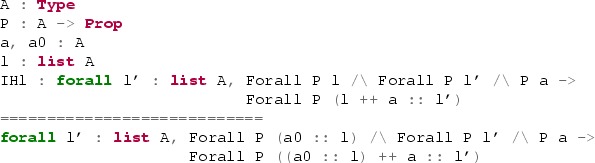
The hammer solves this goal using the lemma Forall_cons, the inductive hypothesis (IHl) and the definition of ++. Note that to reconstruct the ATP proof for this goal it is crucial that our reconstruction tactics can do inversion on inductive predicates in the context.

## Limitations

In this section we briefly discuss the limitations of the current implementation of the CoqHammer tool. We also compare the hammer with the automation tactics already available in Coq.

The intended use of a hammer is to prove relatively simple goals using accessible lemmas. Currently, the hammer works best with lemmas from the Coq standard library. Testing with other libraries has been as yet very limited and the hammer tool may need some adjustments to achieve comparable success rates.

The hammer works best when the goal and the needed lemmas are “close to” first-order logic, as some more sophisticated features of the Coq logic are not translated adequately. In particular, when dependent types are heavily used in a development then the effectiveness of the hammer tool is limited. Specifically, case analysis over inhabitants of small propositional inductive types is not translated properly, and the fact that in Coq all inhabitants of ${\mathrm {Prop}}$ are also inhabitants of ${\mathrm {Type}}$ is not accounted for.

A small propositional inductive type is an inductive type in ${\mathrm {Prop}}$ having just one constructor and whose arguments are all non-informative (e.g. propositional). In Coq it is possible to perform case analysis over an inhabitant of a small propositional inductive type. This is frequently done when dealing with data structures where dependent types are heavily exploited to capture the data structure invariants. Currently, all such pattern matches are translated to a fresh constant about which nothing is assumed. Therefore, the ATPs will fail to find a proof, except for trivial tautologies.

In Coq all propositions (inhabitants of ${\mathrm {Prop}}$) are also types (inhabitants of ${\mathrm {Type}}$). Therefore, type formers expecting types as arguments may sometimes be fed with propositions. For instance, one can use the pair type former as if it was a conjunction. Our translation heavily relies on the possibility of detecting whether a subterm is a proposition or not, in order to translate it to a FOL formula or a FOL term. The currently followed approach to proposition detection is relatively simplistic. For example, the pair type former should be translated to four different definitions, one taking in input two propositions, etc. Currently, only one definition is generated (the one with both arguments being of type ${\mathrm {Type}}$).

In the context of code extraction the above two problems and some similar issues were handled in Pierre Letouzey’s Ph.D. thesis [60]. In [60] Coq terms are translated into an intermediate language where propositions are either removed from the terms or turned into unit types when used as types. It may be worthwhile to investigate if our translation could be factorized reusing the intermediate representation from [60]. If successful, this would be a better approach.

We leave it for future work to increase effectiveness of the hammer on a broader fragment of dependent type theory. In this regard our hammer is similar to hammers for proof assistants based on classical higher-order logic, which are less successful when the goal or the lemmas make heavy use of higher-order features.

The success of the hammer tactic is not guaranteed to be reproducible, because it relies on external ATPs and uses time limits during proof reconstruction. Indeed, small changes in the statement of the goal or a change of hardware may change the behaviour of the hammer. However, once a proof has been found and successfully reconstructed the user should replace the hammer tactic with an appropriate reconstruction tactic shown by the hammer in the response window. This reconstruction tactic does not depend on any time limits or external ATPs, so its success is independent of the current machine.

In comparison to the hammer, domain-specific decision procedures, e.g., the omega tactic, are generally faster and more consistently reliable for the goals they can solve. On the other hand, the proof terms generated by the hammer tactic are typically smaller and contain fewer dependencies which are more human-readable.

An advantage of Coq proof-search tactics like auto, eauto or firstorder is that they can be configured by the user by means of hint databases. However, they are in general much weaker than the hammer. The idea of a hammer is to be a strong general-purpose tactic not requiring much configuration by the user.

## Conclusions and Future Work

We have developed a first whole hammer system for intuitionistic type theory. This involved proposing an approximation of the Calculus of Inductive Constructions, adapting premise selection to this foundation, developing a translation mechanism to untyped-first order logic, and proposing reconstruction mechanisms for the proofs found by the ATPs. We have implemented the hammer as a plugin for the Coq proof assistant and evaluated it on all the proofs in its standard library. The source code of the plugin for Coq versions 8.5, 8.6 and 8.7, as well as all the experiments are available at: http://cl-informatik.uibk.ac.at/cek/coqhammer/

The hammer is able to re-prove completely automatically 40.8% of the standard library proofs on a 8-CPU system in about 40 s. This success rate is already comparable to that offered by the first generations of hammer systems for HOL and Mizar and can already offer a huge saving of human work.

To our knowledge this is the first translation which is usable by hammers. Strictly speaking, our translation is neither sound nor complete. However, our experiments suggest that the encoding is “sound enough” to be usable and that it is particularly good for goals close to first-order logic. Moreover, a “core” version of the translation is in fact sound [27].

There are many ways how the proposed work can be extended. First, the reconstruction mechanism currently is able to re-prove only 85.2% (4215 out of 4841) of the proofs founds by the ATPs, which is lower than that in other systems. The premise selection algorithms are not as precise as those involving machine learning algorithms tailored for particular logics. In particular, for similar size parts of the libraries almost the same premise selection algorithms used in HOLyHammer [52] or Isabelle/MaSh on parts of the Isabelle/HOL library [15], require on average 200–300 best premises to cover the dependencies, whereas in the Coq standard library on average 499–530 best premises are required.

The core of the hammer—the translation to FOL—could be improved to make use of more knowledge available in the prover in order to offer a higher success rate. It could also be modified to make it more effective on developments heavily using dependent types, and to more properly handle the advanced features of the Coq logic, possibly basing on some of the ideas in [60]. Finally, the dependencies extracted from the Coq proof terms do miss information used implicitly by the kernel, and are therefore not as precise as those offered in HOL-based systems.

In our work we have focused on the Coq standard library. Evaluations on a proof assistant standard library were common in many hammer comparisons, however this is rarely the level at which users are actually working, and looking at more advanced Coq libraries could give interesting insights for all components of a hammer. Since we focused on the standard library during development, it is likely that the effectiveness of the hammer is lower on libraries not similar to the standard library.

In particular, the Mathematical Components Library based on SSReflect [37] would be a particularly interesting example, as it heavily relies on unification hints to guide Coq automation. It has been used for example in the proofs of the four color theorem [38] and the odd order theorem [36]. On a few manually evaluated examples, the success rate is currently quite low. It remains to be seen, whether a hammer can provide useful automation also for such developments, and how the currently provided translation could be optimized, to account for the more common use of dependent types. Lastly, we would like to extend the work to other systems based on variants of CIC and other interesting foundations, including Matita, Agda, and Idris.

## Footnotes

1 There are many dependencies among the features, however considering such dependenceis makes premise selection very slow and gives little improvement both when it comes to machine learning metrics and in practical hammer use [4].
